# Influenza A/H3N2 virus infection in immunocompromised ferrets and emergence of antiviral resistance

**DOI:** 10.1371/journal.pone.0200849

**Published:** 2018-07-19

**Authors:** Rueshandra Roosenhoff, Erhard van der Vries, Anne van der Linden, Geert van Amerongen, Koert J. Stittelaar, Saskia L. Smits, Martin Schutten, Ron A. M. Fouchier

**Affiliations:** 1 Department of Viroscience, Erasmus MC, Rotterdam, Zuid- Holland, The Netherlands; 2 Department of Infectious Diseases & Immunology, Division of Virology, Faculty of Veterinary Medicine, Utrecht University, Utrecht, Utrecht, The Netherlands; 3 Viroclinics Biosciences BV, Rotterdam, Zuid-Holland, The Netherlands; 4 Clinical Virology and Diagnostics, Alkmaar, Noord-Holland, The Netherlands; The University of Chicago, UNITED STATES

## Abstract

Influenza viruses can cause severe life threatening infections in high-risk patients, including young children, the elderly and patients with compromised immunity due to underlying medical conditions or immunosuppressive treatment. The impaired immunity of these patients causes prolonged virus infection and combined with antiviral treatment facilitates the emergence of viruses with resistance mutations. The diverse nature of their immune status makes them a challenging group to study the impact of influenza virus infection and the efficacy of antiviral therapy. Immunocompromised ferrets may represent a suitable animal model to assess influenza virus infection and antiviral treatment strategies in immunocompromised hosts. Here, ferrets were given a daily oral solution of mycophenolate mofetil, tacrolimus and prednisolone sodium phosphate to suppress their immune system. Groups of immunocompromised and immunocompetent ferrets were inoculated with an A/H3N2 influenza virus and were subsequently treated with Oseltamivir or left untreated. Quantitative real-time reverse transcription polymerase chain reaction (qRT-PCR) was performed on the throat and nose specimens to study virus replication during the course of infection. All immunocompromised ferrets had prolonged presence of viral RNA and a higher total amount of virus shedding compared to the immunocompetent ferrets. Although Oseltamivir reduced the total amount of virus shedding from the nose and throat of treated ferrets, it also resulted in the emergence of the neuraminidase R292K resistance substitution in all these animals, as determined by mutation specific RT-PCR and next-generation sequencing. No additional mutations that could be associated with the emergence of the R292K resistance mutation were detected. The immunocompromised ferret model can be used to study A/H3N2 virus shedding and is a promising model to study new antiviral strategies and the emergence of antiviral resistance in immunocompromised hosts.

## Introduction

Influenza viruses cause infections in millions of individuals worldwide every year. In most cases, infected patients develop mild clinical symptoms associated with modest virus shedding that usually resolves within one to two weeks. However, high risk groups, including young children, the elderly, pregnant woman and individuals with chronic medical conditions such as obesity, asthma, diabetes and neurological disorders are more likely to develop severe and complicated influenza, with increased risk of mortality [1, 2]. Recent estimates indicated that up to 650,000 people die of respiratory diseases linked to seasonal influenza annually [3]. Moreover, various studies have shown that patients with suppressed immunity due to diseases such as acquired immune deficiency syndrome (AIDS) and autoimmune disorders or due to the use of immunosuppressive drugs e.g. for chemotherapy or solid organ transplantation also have an increased risk to develop serious complications following influenza virus infection [1, 4, 5]. Due to their impaired immune status, they are unable to clear viruses efficiently. Therefore, influenza viruses tend to persist longer and may cause prolonged life threatening infection in these immunocompromised hosts [5–8].

The primary way to prevent influenza virus infection, especially in high-risk groups, is through the use of influenza vaccines [9]. Unfortunately, vaccination is not always fully effective and individuals can still acquire influenza virus infection when the vaccine strains do not sufficiently match with the circulating viruses. Accordingly, antiviral drugs were developed for the treatment of patients with confirmed or suspected influenza virus infection to reduce symptoms and prevent serious complications of influenza [10]. The two classes of antiviral drugs that are available on the market for the treatment of influenza are the matrix protein 2 (M2) ion channel inhibitors amantadine and rimantadine and the neuraminidase (NA) inhibitors (NAI) Oseltamivir, Zanamivir, Peramivir and Laninamivir [11]. Currently, virtually all circulating influenza viruses have resistance substitutions against M2 ion channel inhibitors, whereas the prevalence of resistance substitutions against NAI is low [11, 12]. Therefore, NAI are currently recommended to treat influenza virus infected patients [11, 13].

The overall efficacy of antiviral drugs to resolve clinical symptoms caused by influenza virus infection is relatively modest [14]. Accordingly, new antiviral drugs are being developed that target different steps in the viral replication cycle, such as receptor binding, membrane fusion, and genome transcription or replication, to treat infection with wildtype and resistant influenza viruses [15, 16]. Furthermore, various studies have indicated that combination therapy with different antivirals could be more effective, compared to monotherapy, in inhibiting influenza viruses in immunocompromised patients [17, 18]. The proof of concept for these drugs that are in the pre-clinical and clinical pipeline as well as combination therapy may be demonstrated using appropriate animal models, including models that attempt to capture the situation in immunocompromised patients. It is challenging to assess an optimal regimen of antiviral therapy for immunocompromised patients, due to the heterogeneity of illnesses, varying levels of immune suppression and variable course of virus infection among patients in this group [4, 19]. The majority of randomized clinical trials testing the efficacy of antiviral treatments against influenza virus infection has been conducted in individuals with a competent immune response [14, 20]. The strategies adapted from these studies have also been used to treat influenza virus infected immunocompromised patients and they appear to be effective in these patients [21, 22]. However, virus replication can persist in these patients even after the administration of antiviral treatment [5, 23]. Additionally, it has been reported that immunocompromised patients have a higher risk to acquire antiviral resistance [7, 24, 25]. This may be due to the prolonged infection or the high virus turnover observed in these patients, which provide an increased likelihood of acquiring resistance mutations that are under positive selection in the presence of antiviral treatment.

Ferrets have been widely uses as a model to investigate influenza virus infection, because the susceptibility to infection and the range of clinical signs seen in infected ferrets are very similar to those observed in humans. Moreover, the anatomy of the airways and the distribution patterns of sialic acid receptor expression in the respiratory tract of ferrets are comparable to those in humans [26]. Ferrets may also be an appropriate model to study the levels of virus replication and associated clinical manifestations upon suppression of the immune system. Previous research has shown that ferrets receiving a mixture of immunosuppressive drugs that are also routinely given to humans undergoing solid organ transplantation, had reduced antibody responses following virus infection and deficient lymph node formation, indicating that the immune defense was weakened [5, 27]. It was further shown that, similar to humans, immunocompromised ferrets infected with A/H1N1pdm09 virus had prolonged virus replication compared to immunocompetent ferrets. Treatment of these ferrets with Oseltamivir led to the rapid emergence of viruses with the H275Y NAI resistance substitution in NA [5].

Given that besides A/H1N1pdm09 viruses also A/H3N2 influenza viruses are circulating in nearly every winter season [28, 29], the question arose whether the results observed in immunocompromised ferrets infected with A/H1N1pdm09 would also be applicable to infection with A/H3N2 virus. Therefore, the present study compared the course of infection of A/H3N2 viruses in immunocompetent and immunocompromised ferrets. It was found that immunocompromised ferrets inoculated with A/H3N2 viruses had prolonged presence of viral RNA in the upper and lower respiratory tract and that the treatment of these ferrets with Oseltamivir resulted in the rapid emergence of the resistance substitution R292K in NA. Mutation specific real-time PCR and Illumina next generation sequencing (NGS) revealed that resistance mutations were detected with a low frequency as early as 2 days after inoculation, which later increased over time. Additional minor variants detected by Illumina NGS present in the HA and NA genes were not associated with Oseltamivir treatment. The immunocompromised ferret model for A/H3N2 virus captures several features of influenza virus infection and antiviral drug treatment in immunocompromised humans, and may be useful for future preclinical studies on antiviral drug therapies.

## Material and methods

### Viruses

An Oseltamivir-sensitive influenza A/H3N2 virus A/Netherlands/16/1998 (A/NL/16/98) was used in this study. This virus was isolated from a respiratory specimen upon inoculation of a Madin-Darby Canine Kidney (MDCK) cell culture (van Baalen *et al*. 2014). Biological clones were obtained by plaque purification. A clone with a genome sequence identical to the consensus sequence of the original specimen was used as the inoculum.

### Ferrets

The animal experiment was performed in strict compliance with the European guidelines (EU directive on animal testing 2010/63/EU) and Dutch Legislation (Experiments on Animals Act, 2014). An independent animal experimentation ethical review committee, Stichting Dier Experimenten Commissie Consult, approved the animal studies (EMC-3137; protocol 122-13-13). Eleven-month-old purpose-bred male ferrets were used, which were seronegative for Aleutian disease and circulating influenza viruses (A/H1N1, A/H3N2 and B). The animals were kept in standard housing until the start of the experiment. Four days before inoculation, the animals were transferred to negatively pressured isolator cages at animal biosafety level 3. Animals were sedated during animal handling using a mixture of ketamine (4 to 8 mg/kg) (Henry Schein Animal Health BV, Cuijk, The Netherlands) alone or in combination with medetomidine (0.1 mg/kg) (Henry Schein Animal Health BV, Cuijk, The Netherlands), which was antagonized by the administration of atipamezole (0.1 mg/kg) (Henry Schein Animal Health BV, Cuijk, The Netherlands) afterwards. Euthanasia under anesthesia was performed by exsanguination. Health status and animal behavior was checked twice daily and included the following parameters: weight loss, loss of appetite, degree of gastroenteritis, breathing problems and strong abnormal behavior. Commercial food pellets and water were provided *ad libidum* during the entire course of the experiment.

### Immunosuppressants, antibiotics and antiviral drugs

The following immunosuppressive drugs were used to suppress the immune system of the animals: Mycophenolate mofetil (MMF) powder for infusion (20mg/kg) (CellCept, Roche, Woerden, The Netherlands), tacrolimus concentrate for infusion (5 mg/ml) (Prograft, Astellas Pharma BV, Leiderdorp, The Netherlands) and oral solution of prednisolone sodium phosphate (5 mg/ml) (Hospital Pharmacy, UMCN St Radboud, Nijmegen, The Netherlands). This regimen is similar to the immunosuppressive treatment of patients undergoing solid organ transplantation. To prevent opportunistic infections, all animals received an antibiotic prophylaxis of amoxicillin supplemented with oral suspension of clavulanic acid (250 mg and 62.5 mg per 5 mL, respectively) (Pharmachemie BV, Haarlem, The Netherlands). Oseltamivir phosphate (OSP; 10mg/Kg) was added to the regimes of ferrets receiving antiviral therapy and was provided by Hoffman-La Roche LtD. (Tamiflu, Basel, Switzerland). Preparation of the drugs was done as previous described [5].

### Experimental groups and administration of the drugs

Twenty-four ferrets were randomly assigned into four groups (n = 6) (group 1: immunocompetent, group 2: immunocompromised, group 3: immunocompromised receiving Oseltamivir and group 4: immunocompetent receiving Oseltamivir). A scheme of the experiment is depicted in S1 Fig. At day -4, antibiotics (10 mg/kg amoxicillin and 2.5 mg/kg clavulanic acid) were administered orally once daily to animals in all groups. Starting at day -3, ferrets in groups 2 and 3 were administered a dose of the oral immunosuppressant cocktail (20 mg/kg MMF, 0.5 mg/kg, tacrolimus and 8 mg/kg prednisolone) twice daily. Twenty-four hours after virus inoculation, ferrets in groups 3 and 4 started receiving an oral solution of OSP (10 mg/kg) once daily. Ferrets in groups 1, 2 and 3 were euthanized by exsanguination 18 days post infection. The immunocompetent ferrets in group 4, which received OSP were euthanized on day 7 after inoculation.

### Virology

At day 0, all ferrets were inoculated intranasally and intratracheally with 10^4^ TCID_50_ of A/NL/16/98. Nose and throat swabs were collect daily in 3 ml virus transport medium [30]. RNA was extracted from these samples and virus load was determined using the semi-quantitative real-time reverse transcription polymerase chain reaction (qRT-PCR) targeting the matrix gene segment. Oseltamivir resistance substitutions at amino acid positions 292 (R/K) and 119 (E/V) of NA were monitored using a mutation specific RT-PCR (msRT-PCR) as described [31]. An electron-microscopy-counted influenza A/PR/8/34 virus stock (Advanced Biotechnologies Inc., Maryland, USA) was run in parallel to convert cycle thresholds to virus particle counts (viral RNA copies). Blood samples for serum were taken on day 0 and day 13 post infection. Serum influenza antibody titers against the homologous influenza strain A/Netherlands/16/1998 and the heterologous strain A/Netherlands/271/1995 were determined by hemagglutination inhibition (HI) assay as previously described [32].

### Next generation sequencing

RNA was extracted from throat and nose samples taken 2, 4, 6, 8 and 10 days post inoculation (dpi), from immunocompromised and immunocompetent ferrets treated with Oseltamivir (groups 3 and 4), using the QIAamp Viral RNA Mini Kit (Qiagen, Hilden, Germany). Subsequently, RNA sample volumes were reduced by evaporation in a SpeedVac at 65°C and first-strand cDNA was made using SuperScript III reverse transcriptase (Invitrogen, California, USA) and random primers (ThermoFisher Scientific, Massachusetts, USA) according to the manufacturer’s protocol. Second strand cDNA was synthesized in NEBnext second strand synthesis reaction buffer with second strand enzyme mix (New England Biolabs, Massachusetts, USA) and nuclease-free water. The cDNA was concentrated using the Genomic DNA Clean & concentrator kit (Zymo Research, California, USA) according to the manufacturer’s instructions. The DNA products were quantified using the Qubit dsDNA BR Assay kit (Thermo Fisher Scientific, Massachusetts, USA).

The enrichment, hybridization, Illumina library preparation of the HA and NA gene targets were performed according to the protocol previously described by Eckert *et al*. [33]. Sequencing with a paired-end read length of 300 nucleotides (nt) was conducted on an Illumina MiSeq instrument using the 600-cycle MiSeq Reagent Kit v3 (Illumina, California, USA).

Sequence reads were imported into CLC genomic Workbench version 8 (Qiagen) and trimmed using a quality score cut-off of Q30. The trimmed reads were then mapped against the HA and NA sequences of influenza virus A/Netherlands/5/1998 (GenBank Accession numbers, CY114485 and CY114487). Nucleotide substitutions that resulted in an amino acid substitution relative to the reference sequence of A/Netherlands/5/98 were called if the nucleotide position was covered in more than a 100 reads, with an average quality score of ≥ 30 and a forward and reverse balance of ≥0.4. The substitutions that varied within the samples and had a frequency above 1% were reported in the results tables.

### Statistical analysis

All data is reported as the mean ± standard error of the mean (s.e.m.). The area under the curve (AUC) of the virus load determined by qRT-PCR was calculated for each ferret group using the trapezoid rule with graphpad prism. The student t-test was used to compare AUC values between the groups. Data were considered significant if the *P* value was below 0.05.

## Results

### Prolonged viral RNA presence in immunocompromised ferrets

**Fig 1:**
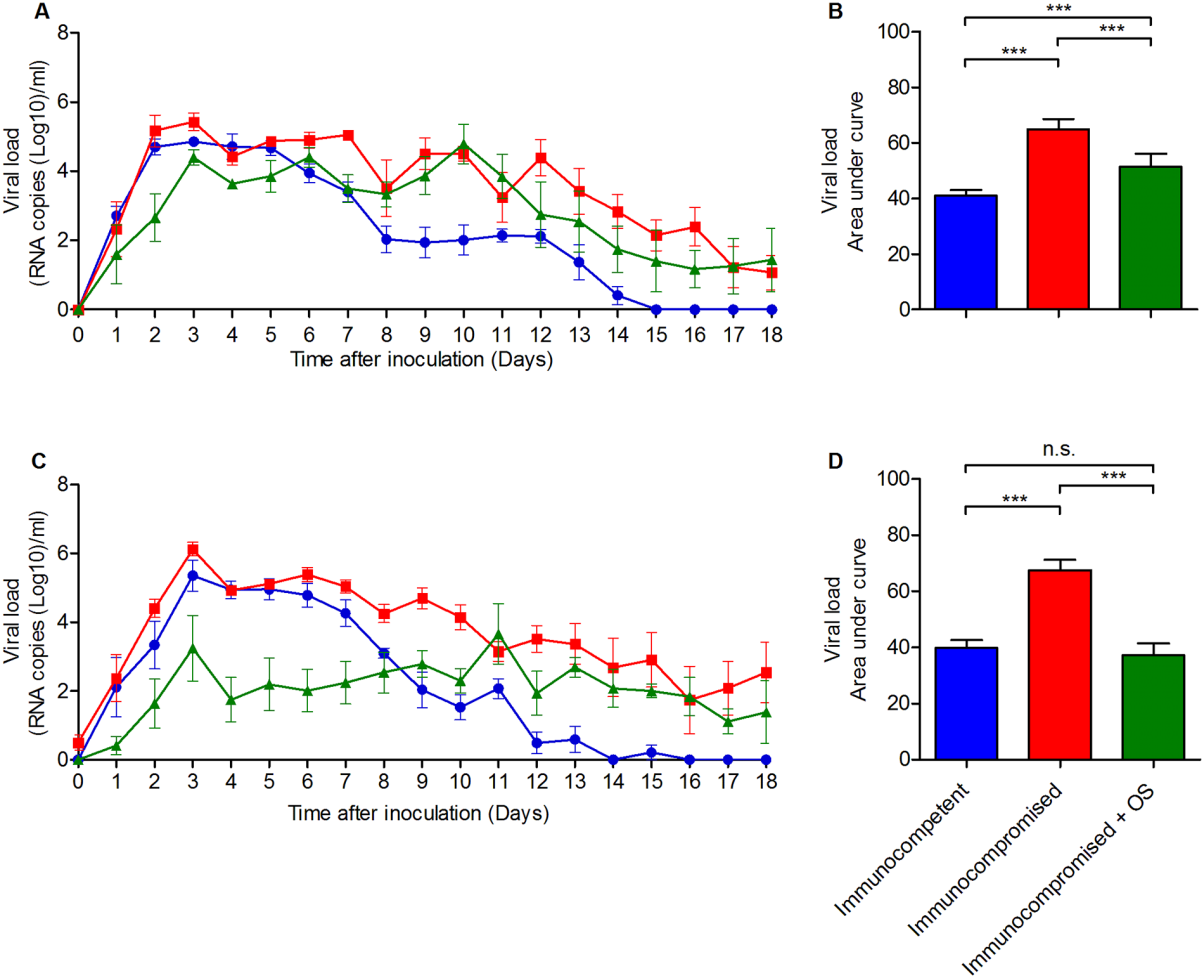
Viral RNA load in ferrets inoculated with influenza virus A/NL/16/98. Immunocompetent (blue) and immunocompromised (red, green) ferrets were inoculated with influenza virus A/NL/16/98 (H3N2) and subsequently treated with Oseltamivir (green) or left untreated (blue, red). Virus RNA load in samples of the throat (A) and nose (C) was determined by qRT-PCR daily for 18 days. The area under the curve of panels A and C was used to estimate the total amount of viral RNA shedding from the throat (B) and nose (D) of the inoculated ferrets. The line and bar graphs depict the mean ± S.E.M. The asterisks indicates a statistically significant P value (***P<0.001).

Ferrets were treated with a cocktail of mycophenolate mofetil, tacrolimus and prednisolone sodium phosphate for 3 days, which was previously shown to cause a steady-state of severe immunosuppression, which was continued by bi-daily dosing until the end of the experiment (S2 Fig) [5, 30]. These immunosuppressed ferrets and immunocompetent control animals were inoculated with 10^4^ TCID_50_ A/NL/16/98 (H3N2) influenza virus. Viral RNA was detected from 1 dpi onwards in the nose and the throat of the ferrets, reaching a peak around 3 dpi (Fig 1A and 1C). In the first week after inoculation, the virus loads were similar between untreated immunocompetent and immunocompromised ferrets. However, immunocompetent ferrets started to clear the infection from 7 dpi onwards with total virus clearance by 14–15 dpi, while immunocompromised ferrets continued to shed viral RNA throughout the course of the experiment (18 days). One group of immunocompromised ferrets inoculated with A/NL/16/98 was treated with Oseltamivir starting at 1 dpi. In this group of ferrets, viral RNA was detected in the throat and nose throughout the 18 days of the experiment, as in ferrets that were not treated with Oseltamivir. The RNA virus load in the throat swabs of immunocompromised ferrets were very similar with or without Oseltamivir treatment while virus loads were substantially lower in the nose swabs of immunocompromised animals treated with Oseltamivir, in particular in the early phase (1–10 dpi) of the infection.

To determine the total amount of viral RNA that was present in the nose and throat swabs of ferrets, the area under the curve (AUC) was calculated for all groups (Fig 1B and 1D). The total amount of virus shedding from both the throat and the nose of untreated immunocompromised ferrets was significantly higher as compared to the throat and nose of immunocompetent ferrets (P<0.001). Treatment of immunocompromised ferrets with Oseltamivir resulted in significantly lower virus shedding from both the nose and throat compared to untreated immunocompromised ferrets. The reduction of virus shedding from the throat of immunocompromised ferrets as a consequence of Oseltamivir treatment was relatively modest as compared to the reduction of virus shedding from the nose.

In a separate experiment, immunocompetent ferrets inoculated with influenza virus A/NL/16/98 were also treated with Oseltamivir, but only for 7 days. The levels of virus shedding from the nose and throat of these animals were similar to those of immunocompromised ferrets treated with Oseltamivir, as were the total amounts of viral RNA as calculated from the AUC (S3 Fig).

### Emergence of Oseltamivir resistant viruses in immunocompromised ferrets treated with Oseltamivir

**Fig 2:**
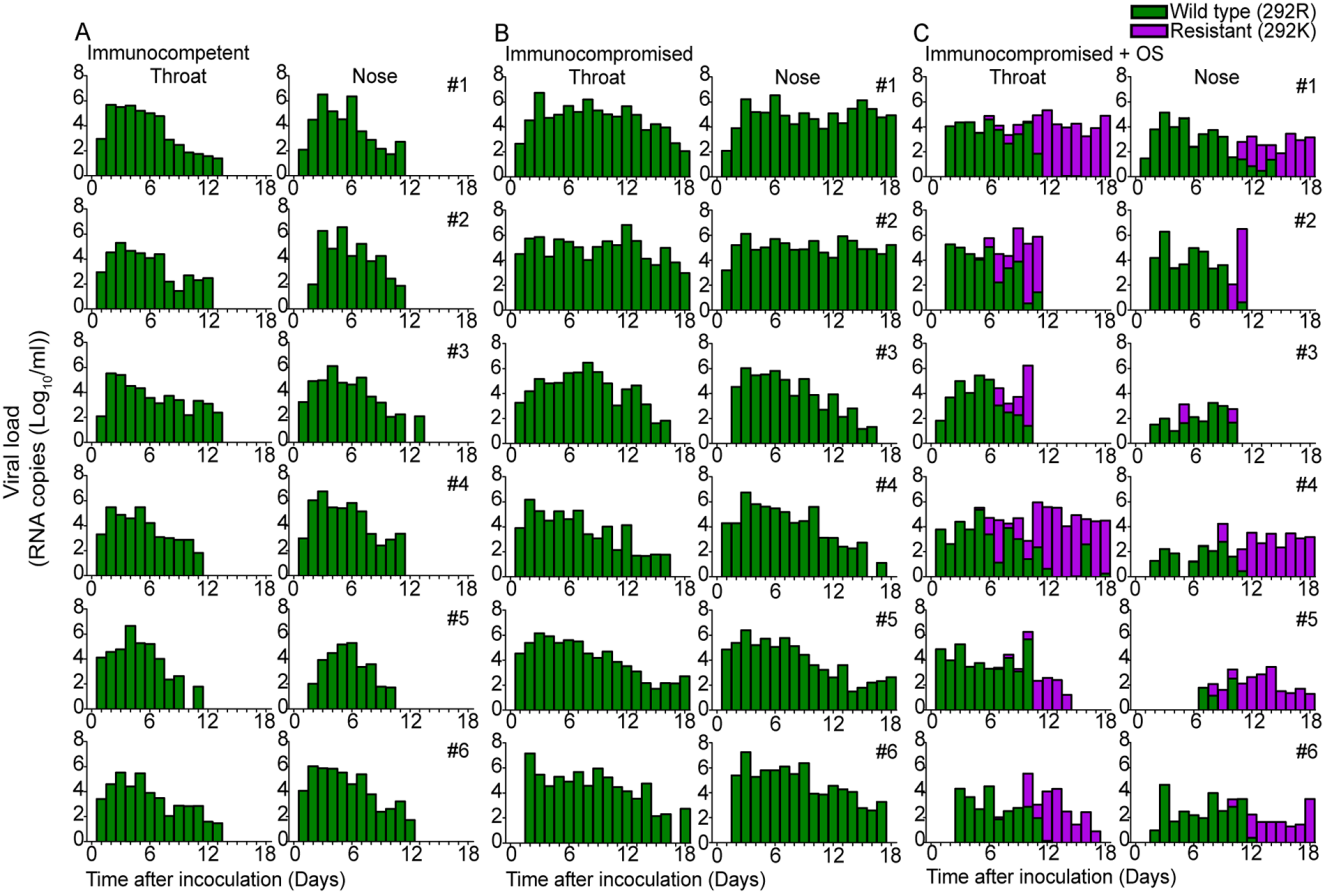
Emergence of Oseltamivir resistant viruses in ferrets. The emergence of Oseltamivir resistant viruses was monitored in throat and nose samples from untreated immunocompetent ferrets (A), untreated immunocompromised ferrets (B) and Oseltamivir treated immunocompromised ferrets (C) by mutation-specific RT-PCR for the Oseltamivir-resistance mutation R292K. The green bars depict the presence of the wildtype genotype (292R) and the magenta the presence of the resistant genotype (292K). If both genotypes were present in one sample, the proportions of the genotypes are stacked.

Samples from all inoculated animals (Fig 2 and S3 Fig) were analyzed by mutation-specific RT-PCR to detect substitutions E119V and R292K in NA, the most common Oseltamivir associated resistance mutations in A/H3N2 influenza viruses [34]. Mutation E119V was not detected in these samples, whereas the R292K resistance mutation was detected in the nose and throat swabs of all immunocompromised (Fig 2C) ferrets that were treated with Oseltamivir. In contrast, emergence of viruses with resistance mutations did not occur in any of the untreated immunocompetent and immunocompromised ferrets, indicating that resistance mutations did not emerge spontaneously. The R292K substitution first emerged in the throat at 5dpi in one animal (ferret 4) and at 6dpi and 7dpi in three (ferret 1, 2, 3) and two animals (ferret 5 and 6) respectively. When the R292K substitution occurred, it was initially present as a minor genotype in a mixed population of wild type and resistant viruses. At later time points, viruses with the resistance mutation represented the major genotype and frequently even the only genotype in the virus population (Fig 2C). In two immunocompromised ferrets that were treated with Oseltamivir, virus was cleared from the nose and throat by 10–11 dpi. However, there was no difference in the emergence of Oseltamivir resistance mutations in viruses of animals that did (ferret 2 and 3) or did not (ferret 1, 4, 5, 6) clear the virus earlier (Fig 2C). There were no substantial differences in the kinetics of emergence of Oseltamivir resistance mutations in immunocompetent ferrets treated with Oseltamivir as compared to immunocompromised ferrets, although resistant viruses were not detected in one of the animals in this short time-course experiment (S4 Fig).

### Lack of detection of additional mutations associated with Oseltamivir treatment

**Fig 3:**
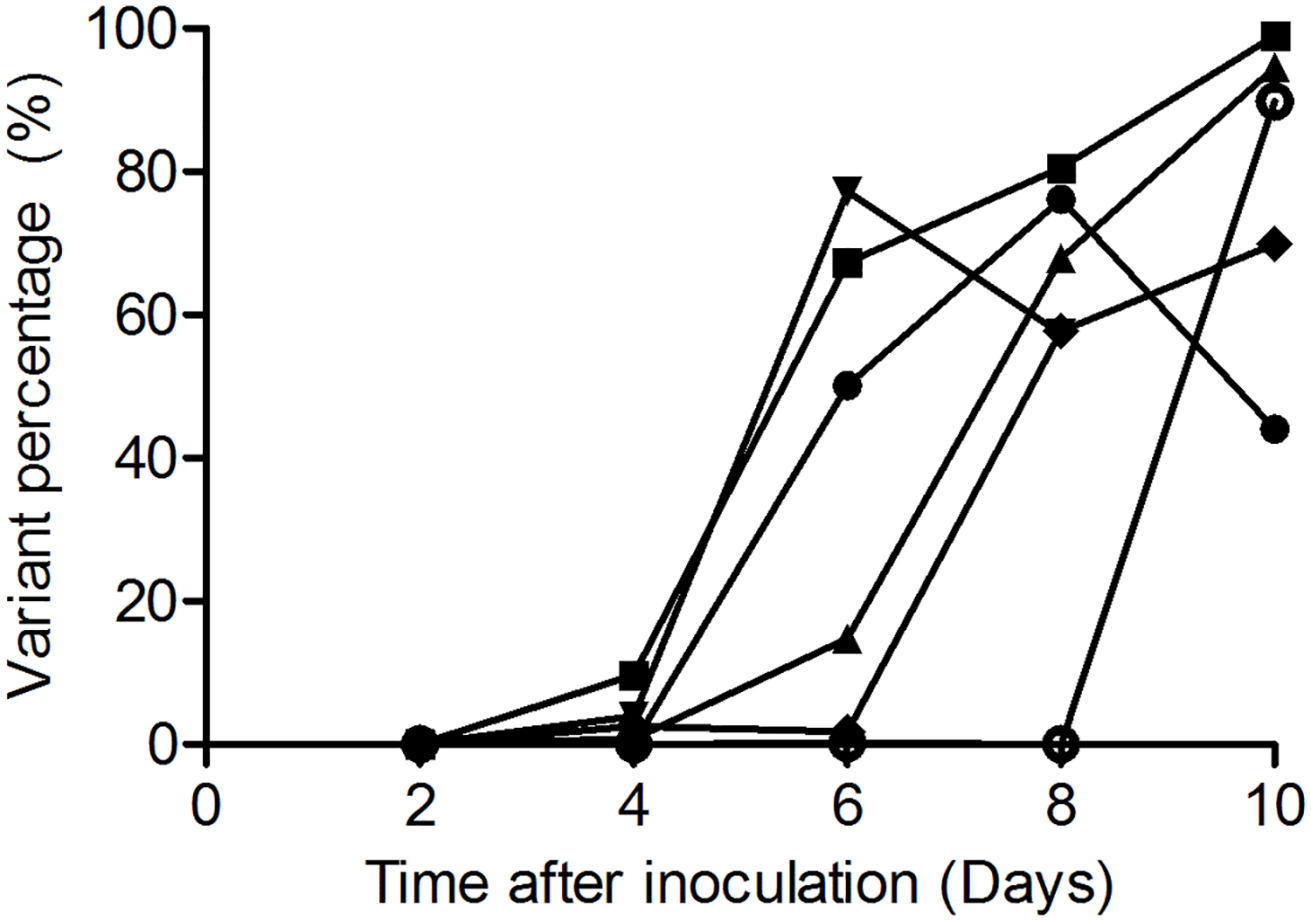
The frequency of the R292K mutation as determined by Illumina NGS in Oseltamivir treated immunocompromised ferrets. The percentage of the R292K variant was determined in pooled throat/nose samples collected at 2, 4, 6, 8 and 10 dpi from immunocompromised ferrets that received Oseltamivir treatment. The graph depicts the frequency of the R292K mutation over time for each ferret separately, with circles, squares, triangles, down triangles, diamonds and open circles representing ferrets 1–6 respectively.

Illumina NGS was conducted to analyze the emergence of mutations beyond R292K that were potentially associated with Oseltamivir treatment in the HA and NA genes of influenza viruses in immunocompetent and immunocompromised ferrets at 2, 4, 6, 8 and 10 dpi. To validate this approach, the frequency of the R292K resistance substitutions was first determined by Illumina NGS. There was good overall correspondence between the relative proportion of the R292K resistance substitution as determined by msRT-PCR and Illumina NGS (S5 Fig, R^2^ = 0.94, P<0.0001). For some ferrets, the resistance mutation was already detected at 4 dpi at a low frequency by Illumina NGS, when it was not yet detected by msRT-PCR (S5 Fig). Like the msRT-PCR analysis, Illumina NGS allowed the detection of R292K in all immunocompromised and immunocompetent ferrets treated with Oseltamivir (Fig 3 and S6 Fig) (Table 1 and S1 Table).

**Table 1:** Amino acid substitution in hemagglutinin and neuraminidase sequences of OS treated immunocompromised ferrets by Illumina next generation sequencing.

Ferret	Day^a^	R292K (%)	AA^b^ change in HA^b^ (%)	AA change in NA^b^ (%)
**1**	2	0		
	4	0	L29P(2.1), A85T(8.3), F516L(2.5)	V424G(1.0)
	6	49.9	D120N(1.1), V418F(1.1)	I26T(1.5), V30A(5.7), V33A(2.0), Y40H(2.6), C42R(2.6), V50A(2.3), I57T(2.2), I62T(1.9), L140I(1.4)
	8	76.2		N329T(1.5)
	10	44.1	F15S(1.6), N440K(1.2)	N47D(1.8), **T242I(25.0)**
**2**	2	0		T34I(1.3)
	4	9.6	S281R(1.9), M284I(1.2), D287A(1.2), F348L(1.3), E442G(1.5)	P46L(1.0), G286D(1.4)
	6	67.2	K308N(1.0)	
	8	80.5		P126S(1.4)
	10	98.9		
**3**	2	0		P285L(1.8)
	4	0.8	G132D(1.3), R508G(1.4)	
	6	14.8	S165N(1.0), S225R(2.5), I246M (4.4), S263N(4.8), F274S(1.8), I276T(1.1), R323K(3.9), E442G(1.8)	
	8	67.9	T26(7.3), D69N(1.1), N205I(1.2), A214V(1.4), I242F(1.3), A320V(1.1), A350T(1.1)	
	10	94.5	F95L(1.4), **T151A(25.2)**	E83K(1.0), I222N(1.4)
**4**	2	0		
	4	4.0	T386I(2.0), M478I(1.0)	I28V(4.6), T34A(3.0), L52M(2.0)
	6	77.1	A6S(1.0), F536L(1.5), F550S(2.5)	I62T(1.1), T69N(1.1), D309G(1.5)
	8	57.6	F95L(2.2), T151A(1.3)	D83K(1.2)
	10	0		
**5**	2	0	D120N(1.5), A537V(1.9)	
	4	2.6	C30R(2.6), K403N(1.5)	
	6	1.8	G21R(6.6), G21V(3.2), S111G(6.9), E442G(5.7), L463Q(2.0)	
	8	57.7	H200P(1.1)	N208S(1.2)
	10	69.7	A320P(1.3), L443F(1.0)	
**6**	2	0		
	4	0	P324S(1.8), E419G(5.3)	T195A(1.5), T242I(1.9), D330E(3.8)
	6	0	P119A(1.6), R472K(1.5)	
	8	0		
	10	89.8	N232S(1.6)	

^a^ Time after inoculation when resistance mutations is detected.

^b^ Abbreviation: AA, amino acid; HA, hemagglutinin; NA, neuraminidase.

Mutations with a frequency above 20% are marked in bold.

Illumina NGS revealed numerous mutations in HA and NA of the viruses beyond R292K, present in all Oseltamivir treated animals (Table 1, S1 Table). Minor variants with frequencies ranging from 1.0–7.7% were detected in the HA or NA genes in samples from all ferrets but one. In some ferret samples, such HA and NA mutations became predominant, but only temporarily. Examples of substitutions are NA-T242I (ferret 1 at 10 dpi) and HA-T151A (ferret 3 at 10 dpi) (Table 1). However, where viruses with the R292K substitution in NA generally increased in prevalence throughout the course of the experiment, to become dominant at the latest time points, none of the additional substitutions followed a similar trend. Thus, under these experimental conditions, the emergence of viruses with the R292K substitution in NA was not accompanied by co-mutations in NA and HA that could act to compensate for potential fitness losses associated with Oseltamivir resistance.

## Discussion

Here, the immunocompromised ferret model for influenza A virus infection and Oseltamivir treatment that was recently described for A/H1N1pdm09 virus infection [5] was evaluated for infection with an influenza A/H3N2 virus. The immune suppressive regimen employed in this immunocompromised ferret model mimics the regimen used for solid organ transplant recipients. Like A/H1N1pdm09 influenza virus, influenza A/H3N2 viruses continue to cause annual epidemics with substantial morbidity and mortality, for which NAI are used routinely as antiviral drugs, in particular in individuals with an impaired immune response.

Although the level of immunosuppression was not addressed directly in the present study, in previous studies it was shown that this ferret model results in immunosuppression as evidenced by lower influenza specific HI antibody titers, impaired formation of the lymphoid follicles present in the tracheobronchial lymph nodes and depletion of lymphocytes in the tonsils of the ferrets [5]. Moreover, it was shown that the immunocompromised ferrets had sustained A/H1N1pdm09 virus shedding compared to the immunocompetent ferrets. Accordingly, similar to influenza virus infection in immunocompromised patients, the present data showed that influenza A/H3N2 viral RNA was detected for a longer period in immunocompromised ferrets compared to the immunocompetent ferrets [5, 8]. Apparently, the immune response of the ferrets receiving the immunosuppressive drugs was not effective in clearing the virus infection, leading to more prolonged virus presence in the immunocompromised host. This indicated that immunocompromised ferrets might represent a suitable model to analyze virus shedding in immunocompromised hosts.

The effect of NAI such as Oseltamivir against influenza virus infection in immunocompromised patients has been controversial, where some clinical studies showed that Oseltamivir was effective to reduce disease burden, viral shedding, symptom severity, length of hospital stay and mortality, while others claimed that it had no or minimum beneficial effects [14, 35–39]. The current data showed that the treatment of both immunocompromised and immunocompetent ferrets with Oseltamivir resulted in a lower total A/H3N2 viral RNA load in the nose and the throat samples compared to the ferrets that were untreated [6]. Interestingly, this reduction of viral RNA in Oseltamivir treated animals was larger in the nose compared to the throat. Oseltamivir has been shown to reduce viral replication in the nose of influenza A/H5N1 and A/H1N1pdm09 infected ferrets [40–42]. However, throat samples were not taken from the ferrets in these studies. Therefore, it is not known whether the reduction of viral replication in the upper respiratory tract was higher compared to that in the lower respiratory tract upon Oseltamivir treatment of ferrets, which is largely reflected by viral replication in the nose and throat swabs respectively. The difference seen in total viral RNA load in the nose and throat upon Oseltamivir treatment could be due to a difference in the Oseltamivir concentration that is present in these areas of the respiratory tract. Additionally, since viral RNA loads peaked earlier in the throat of the ferrets compared to the nose, it is also possible that Oseltamivir treatment has to accommodate with higher amounts of virus compared to the nose, as studies have shown that virus inhibition by Oseltamivir is less efficient at higher virus titers [40, 43, 44]. Therefore, the distribution of Oseltamivir throughout the respiratory tract of the ferret is essential to reduce viral replication efficiently, which remains to be elucidated. Even though there was a reduction in total viral RNA upon Oseltamivir treatment, this did not lead to complete viral clearance, as viral RNA was still present at 18 dpi in immunocompromised ferrets. This was in accordance with a previous study demonstrating that immunocompromised ferrets inoculated with A/H1N1pdm09 still had prolonged viral shedding upon treatment with Oseltamivir [5]. In the present study, none of the ferrets died during the course of the experiment or developed severe disease, hence no conclusions can be drawn about the effectiveness of Oseltamivir in reducing morbidity and mortality. In the previous study with A/H1N1pdm09 virus, Oseltamivir treatment was found to decrease mortality in immunocompromised ferrets [5].

Oseltamivir treatment led to the emergence the R292K substitution in immunocompromised and immunocompetent ferrets inoculated with A/H3N2 virus. The resistant R292K genotype of the A/H3N2 virus was detected initially as a minor variant by msRT-PCR. With Illumina NGS, resistance mutation R292K was already detected at 4 dpi in Oseltamivir treated ferrets. None of the immunocompetent and immunocompromised ferrets had acquired the E119V, although this is also a common mutation associated with Oseltamivir treatment that is found in NA. The E119V mutation was found to cause a 20 to 1000-fold increase in the 50% inhibitory concentration (IC_50_) of Oseltamivir [45, 46]. The R292K substitution was found to cause a greater reduction in susceptibility to Oseltamivir compared to the E119V substitution, conferring a > 9000 increase of the Oseltamivir IC_50_ [45, 46]. Plasma levels of Oseltamivir have been shown to reach approximate concentrations between 2561 and 9603nM in ferrets [5], which is higher than the IC_50_ associated with the E119V mutation. This could explain why the E119V mutation was not detected in the present study.

Previous studies have shown that the emergence of resistance mutations impaired the fitness of influenza viruses but that additional mutations may be acquired in HA or NA to compensate for this fitness loss [47–49]. This is relevant as the prolonged viral shedding in immunocompromised hosts could allow expansion of the quasi-species of influenza viruses present in the host due to the error prone viral replication. This in combination with Oseltamivir treatment creates a strong selective pressure on the influenza virus variant population and facilitates the emergence of resistant virus as a dominant variant in the population, if needed upon acquisition of additional–compensatory–mutations. In immunocompetent patients the resistant viruses may be cleared before they acquire such compensatory substitution. The presence of such resistant viruses that have regained full viral fitness may negatively impact the efficacy of the Oseltamivir treatment and account for the sustained virus shedding in the ferrets, even after the reduction of the total virus load. Three immunocompromised ferrets in the present study acquired additional substitutions in the HA (T151A) and the NA (T242I) proteins besides the R292K resistance substitution, which could represent compensatory changes. The substitution T151A in HA is not common in circulating viruses in the human population, as it is not present in the HA sequences in the Global Initiative on Sharing All Influenza Data (GISAID) Epiflu database. The T242I substitution in NA has previously only been detected in an immunocompromised child infected with multidrug resistant A/H3N2 virus [24]. The substitution was present together with the known E119V and I222V resistance substitutions. Therefore, this substitution may be associated with antiviral treatment and might have a compensatory function. However, in the immunocompromised child it was only detected in one clinical sample and did not become fixed in the virus population, suggesting that it may have emerged by chance and was not involved in determining antiviral resistance. Future experiments may be conducted to study the function of the substitution on the virus phenotype by reverse genetics. The other substitutions in HA (T151A) and NA (I57T) have not been identified in previous studies and their functions remain unknown.

Individuals with suppressed immunity due to age, pregnancy, disease or treatment with immunosuppressive drugs are susceptible to various virus infections beyond influenza, which can lead to high morbidity and mortality [4, 7, 16, 50]. The search for ideal medications to treat these various viral infections in immunocompromised patients will lead to the same challenges as faced with the development of anti-influenza viral treatments. The immunocompromised ferret model may be further developed to analyze other viral infections that occur in immunocompromised patients and to design novel drugs to treat them. For instance, Stittelaar *et al*. demonstrated that ferrets with a suppressed immunity were susceptible to human respiratory syncytial virus (HRSV) infection and that the virological outcome observed in these ferrets mimicked the increased virus loads and sustained virus infection seen in humans [27]. Moreover, they showed that the prophylactic treatment of HRSV-infected immunocompromised ferrets with Palivizumab temporarily reduced virus replication, but it resulted in increased virus load (25). Thus, the immunocompromised ferret model is a suitable model to evaluate virus infections in immunocompromised hosts as well as specific intervention strategies for these viruses.

In conclusion, the present study demonstrated that the immunocompromised ferret model can be used to study virus shedding of influenza A/H3N2 virus. The immunocompromised ferret model is a promising model to study the duration of virus shedding and the efficacy of novel mono and combination antiviral treatments for immunocompromised humans. In combination with msRT-PCR and Illumina NGS, this model may also be used to evaluate the chance of developing and selecting mutations associated with drug resistance, direct and compensatory, in immunocompromised hosts.

## Supporting information

S1 Fig Scheme of time of treatment start and virus inoculation in ferrets of the 4 experimental groups. Four days before virus inoculation, all ferrets were given a cocktail of antibiotics (10 mg/kg amoxicillin and 2.5 mg/kg clavulanic acid) once daily till the end of the experiment. The ferrets assigned to the immunosuppressive groups (group 2 and 3) were given a mixture of immunosuppressant (20 mg/kg MMF, 0.5 mg/kg, tacrolimus and 8 mg/kg prednisolone) starting 3 days prior to virus inoculation twice daily till the end of the experiment. All ferrets were inoculated on day 0 with 10^4^ TCID50 A/NL/16/98 (H3N2) influenza virus. One day after virus infection, OSP (10 mg/kg) was administered to ferrets from group 3 and 4. Ferrets from groups 1, 2 and 3 were euthanized 18 days post infection and the once from group 4 were euthanized 7 days post infection.
(PDF)

S2 Fig Influenza antibody titers of immunocompetent and immunocompromised ferrets against influenza viruses A/NL/16/98 and A/NL/271/95. Serum influenza antibody titers of the untreated immunocompetent and immunocompromised ferrets against the influenza homologous strain (A) A/NL/16/98 and (B) the heterologous strain A/NL/271/95 of immunocompetent and immunocompromised ferrets were determined by hemagglutinin inhibition (HI) assay. The antibody titer of each animal are depicted as individuals points. The horizontal bars represent the mean ± S.E.M.
(PDF)

S3 Fig Viral RNA load in ferrets inoculated with influenza virus A/NL/16/98. Immunocompetent (blue, purple) and immunocompromised (red, green) ferrets were inoculated with influenza virus A/NL/16/98 (H3N2) and subsequently treated with oseltamivir (green, purple) or left untreated (blue, red). Virus RNA load in samples of the throat (A) and nose (C) was determined by qRT-PCR daily for 7 days. The area under the curve of panels A and C was used to estimate the total amount of viral RNA shedding from the throat (B) and nose (D) of the inoculated ferrets. The line and bar graphs depict the mean ± S.E.M. The asterisks indicates a statistically significant P value (0.01<**P>0.001, ***P<0.001).
(PDF)

S4 Fig Emergence of oseltamivir resistant viruses in ferrets. The emergence of oseltamivir resistant viruses was monitored in throat and nose samples from untreated immunocompetent ferrets (A), untreated immunocompromised ferrets (B), oseltamivir treated immunocompromised ferrets (C) and treated immunocompetent ferrets (D) by mutation-specific RT-PCR for the oseltamivir-resistance mutation R292K. The green bars depict the presence of the wildtype genotype (292R) and the magenta the presence of the resistant genotype (292K). If both genotypes were present in one sample, the proportions of the genotypes are stacked.
(PDF)

S5 Fig Correspondence between the R292K resistance mutation as measured by mutation specific RT-PCR and Illumina next generation sequencing. The relative proportion of the R292K resistance mutation detected in samples of all oseltamivir treated immunocompromised (grey circles) and immunocompetent (open squares) ferrets inoculated with A/NL/16/98 (H3N2) as determined by mutation specific RT-PCR was compared to the relative proportion determined by Illumina NGS.
(PDF)

S6 Fig The frequency of the R292K mutation as determine by Illumina next generation sequencing in oseltamivir treated immunocompetent ferrets. The percentage of the R292K variant was determined in pooled throat/nose samples collected at 2, 4, 6, 8 and 10 dpi from immunocompetent ferrets that received oseltamivir treatment. The graph depicts the frequency of the R292K mutation over time for each ferret separately, with circles, squares, triangles, down triangles, diamonds and open circles representing ferrets 1–6 respectively.
(PDF)

S1 Table Amino acid substitution in hemagglutinin and neuraminidase sequences of OS treated immunocompetent ferrets by Illumina next-generation sequencing. (PDF)

S1 File ARRIVE guidelines checklist. (PDF)

## References

[1] Fraaij PL, Schutten M, Javouhey E, Burleigh L, Outlaw R, Kumar D, et al Viral shedding and susceptibility to oseltamivir in hospitalized immunocompromised patients with influenza in the Influenza Resistance Information Study (IRIS). Antiviral therapy. 2015;20(6):633–42. 10.3851/IMP2957 25849228

[2] Writing Committee of the WHOCoCAoPI, Bautista E, Chotpitayasunondh T, Gao Z, Harper SA, Shaw M, et al Clinical aspects of pandemic 2009 influenza A (H1N1) virus infection. The New England journal of medicine. 2010;362(18):1708–19. 10.1056/NEJMra1000449 20445182

[3] Organization WH. WHO | Up to 650 000 people die of respiratory diseases linked to seasonal flu each year: World Health Organization; 2017 [updated 2017-12-15 17:46:45. http://www.who.int/mediacentre/news/releases/2017/seasonal-flu/en/.

[4] Kunisaki KM, Janoff EN. Influenza in Immunosuppressed Populations: A Review of Infection Frequency, Morbidity, Mortality, and Vaccine Responses. The Lancet Infectious diseases. 2009;9(8):493–504. 10.1016/S1473-3099(09)70175-6 19628174 PMC2775097

[5] van der Vries E, Stittelaar KJ, van Amerongen G, Veldhuis Kroeze EJ, de Waal L, Fraaij PL, et al Prolonged influenza virus shedding and emergence of antiviral resistance in immunocompromised patients and ferrets. PLoS Pathog. 2013;9(5):e1003343 10.1371/journal.ppat.1003343 23717200 PMC3662664

[6] Lee N, Chan PK, Hui DS, Rainer TH, Wong E, Choi KW, et al Viral loads and duration of viral shedding in adult patients hospitalized with influenza. The Journal of infectious diseases. 2009;200(4):492–500. 10.1086/600383 19591575 PMC7110250

[7] Memoli MJ, Athota R, Reed S, Czajkowski L, Bristol T, Proudfoot K, et al The natural history of influenza infection in the severely immunocompromised vs nonimmunocompromised hosts. Clin Infect Dis. 2014;58(2):214–24. 10.1093/cid/cit725 24186906 PMC3871797

[8] Fleury H, Burrel S, Balick Weber C, Hadrien R, Blanco P, Cazanave C, et al Prolonged shedding of influenza A(H1N1)v virus: two case reports from France 2009. Euro Surveill. 2009;14(49). 20003906

[9] Grohskopf LA, Sokolow LZ, Broder KR, Walter EB, Bresee JS, Fry AM, et al Prevention and Control of Seasonal Influenza with Vaccines: Recommendations of the Advisory Committee on Immunization Practices—United States, 2017–18 Influenza Season. MMWR Recommendations and reports: Morbidity and mortality weekly report Recommendations and reports. 2017;66(2):1–20. 10.15585/mmwr.rr6602a1 28841201 PMC5837399

[10] Fiore AE, Fry A, Shay D, Gubareva L, Bresee JS, Uyeki TM. Antiviral agents for the treatment and chemoprophylaxis of influenza—recommendations of the Advisory Committee on Immunization Practices (ACIP). MMWR Recommendations and reports: Morbidity and mortality weekly report Recommendations and reports. 2011;60(1):1–24. 21248682

[11] Ison MG. Antivirals and resistance: influenza virus. Curr Opin Virol. 2011;1(6):563–73. 10.1016/j.coviro.2011.09.002 22440914

[12] Whitley RJ, Boucher CA, Lina B, Nguyen-Van-Tam JS, Osterhaus A, Schutten M, et al Global assessment of resistance to neuraminidase inhibitors, 2008–2011: the Influenza Resistance Information Study (IRIS). Clin Infect Dis. 2013;56(9):1197–205. 10.1093/cid/cis1220 23307766

[13] Yoo JW, Choi SH, Huh JW, Lim CM, Koh Y, Hong SB. Peramivir is as effective as oral oseltamivir in the treatment of severe seasonal influenza. J Med Virol. 2015;87(10):1649–55. 10.1002/jmv.24232 25946636

[14] Jefferson T, Jones MA, Doshi P, Del Mar CB, Hama R, Thompson MJ, et al Neuraminidase inhibitors for preventing and treating influenza in healthy adults and children. The Cochrane database of systematic reviews. 2014(4):Cd008965 10.1002/14651858.CD008965.pub4 22258996

[15] Koszalka P, Tilmanis D, Hurt AC. Influenza antivirals currently in late-phase clinical trial. Influenza Other Respir Viruses. 2017;11(3):240–6. 10.1111/irv.12446 28146320 PMC5410715

[16] Waghmare A, Englund JA, Boeckh M. How I treat respiratory viral infections in the setting of intensive chemotherapy or hematopoietic cell transplantation. Blood. 2016;127(22):2682–92. 10.1182/blood-2016-01-634873 26968533 PMC4891952

[17] Meijer WJ, Kromdijk W, van den Broek MP, Haas PJ, Minnema MC, Boucher CA, et al Treatment of Immunocompromised, Critically Ill Patients with Influenza A H1N1 Infection with a Combination of Oseltamivir, Amantadine, and Zanamivir. Case Rep Infect Dis. 2015;2015:504975 10.1155/2015/504975 26346659 PMC4546743

[18] Dunning J, Baillie JK, Cao B, Hayden FG, International Severe Acute R, Emerging Infection C. Antiviral combinations for severe influenza. The Lancet Infectious diseases. 2014;14(12):1259–70. 10.1016/S1473-3099(14)70821-7 25213733 PMC7164787

[19] Ison MG, de Jong MD, Gilligan KJ, Higgs ES, Pavia AT, Pierson J, et al End points for testing influenza antiviral treatments for patients at high risk of severe and life-threatening disease. The Journal of infectious diseases. 2010;201(11):1654–62. 10.1086/652498 20423224 PMC12821767

[20] Duval X, van der Werf S, Blanchon T, Mosnier A, Bouscambert-Duchamp M, Tibi A, et al Efficacy of oseltamivir-zanamivir combination compared to each monotherapy for seasonal influenza: a randomized placebo-controlled trial. PLoS Med. 2010;7(11):e1000362 10.1371/journal.pmed.1000362 21072246 PMC2970549

[21] Kumar D, Michaels MG, Morris MI, Green M, Avery RK, Liu C, et al Outcomes from pandemic influenza A H1N1 infection in recipients of solid-organ transplants: a multicentre cohort study. The Lancet Infectious diseases. 2010;10(8):521–6. 10.1016/S1473-3099(10)70133-X 20620116 PMC3045703

[22] Choi SM, Boudreault AA, Xie H, Englund JA, Corey L, Boeckh M. Differences in clinical outcomes after 2009 influenza A/H1N1 and seasonal influenza among hematopoietic cell transplant recipients. Blood. 2011;117(19):5050–6. 10.1182/blood-2010-11-319186 21372154 PMC3109531

[23] Speers DJ, Williams SH, Pinder M, Moody HR, Hurt AC, Smith DW. Oseltamivir-resistant pandemic (H1N1) 2009 influenza in a severely ill patient: the first Australian case. Med J Aust. 2010;192(3):166–8. 20121687 10.5694/j.1326-5377.2010.tb03459.x

[24] Baz M, Abed Y, McDonald J, Boivin G. Characterization of multidrug-resistant influenza A/H3N2 viruses shed during 1 year by an immunocompromised child. Clin Infect Dis. 2006;43(12):1555–61. 10.1086/508777 17109288

[25] Hurt AC, Chotpitayasunondh T, Cox NJ, Daniels R, Fry AM, Gubareva LV, et al Antiviral resistance during the 2009 influenza A H1N1 pandemic: public health, laboratory, and clinical perspectives. The Lancet Infectious diseases. 2012;12(3):240–8. 10.1016/S1473-3099(11)70318-8 22186145

[26] Moore IN, Lamirande EW, Paskel M, Donahue D, Kenney H, Qin J, et al Severity of clinical disease and pathology in ferrets experimentally infected with influenza viruses is influenced by inoculum volume. J Virol. 2014;88(23):13879–91. 10.1128/JVI.02341-14 25187553 PMC4248961

[27] Stittelaar KJ, de Waal L, van Amerongen G, Veldhuis Kroeze EJ, Fraaij PL, van Baalen CA, et al Ferrets as a Novel Animal Model for Studying Human Respiratory Syncytial Virus Infections in Immunocompetent and Immunocompromised Hosts. Viruses. 2016;8(6). 10.3390/v8060168 PMC4926188 27314379

[28] Organization WH. WHO | Influenza update—298 World Health Organization 2017 [updated 2017-09-20 12:56:57. http://www.who.int/influenza/surveillance_monitoring/updates/latest_update_GIP_surveillance/en/.

[29] Suzuki Y. Selecting vaccine strains for H3N2 human influenza A virus. Meta Gene. 2015;4:64–72. 10.1016/j.mgene.2015.03.003 25893173 PMC4392175

[30] van den Brand JM, Stittelaar KJ, van Amerongen G, Reperant L, de Waal L, Osterhaus AD, et al Comparison of temporal and spatial dynamics of seasonal H3N2, pandemic H1N1 and highly pathogenic avian influenza H5N1 virus infections in ferrets. PLoS One. 2012;7(8):e42343 10.1371/journal.pone.0042343 22905124 PMC3414522

[31] van der Vries E, Anber J, van der Linden A, Wu Y, Maaskant J, Stadhouders R, et al Molecular assays for quantitative and qualitative detection of influenza virus and oseltamivir resistance mutations. The Journal of molecular diagnostics: JMD. 2013;15(3):347–54. 10.1016/j.jmoldx.2012.11.007 23597879

[32] Koel BF, Mögling R, Chutinimitkul S, Fraaij PL, Burke DF, van der Vliet S, et al Identification of Amino Acid Substitutions Supporting Antigenic Change of Influenza A(H1N1)pdm09 Viruses. Journal of virology. 2015;89(7):3763–75. 10.1128/JVI.02962-14 25609810 PMC4403388

[33] Eckert SE, Chan JZ, Houniet D, consortium P, Breuer J, Speight G. Enrichment by hybridisation of long DNA fragments for Nanopore sequencing. Microb Genom. 2016;2(9):e000087 10.1099/mgen.0.000087 28785419 PMC5537632

[34] Nguyen HT, Fry AM, Gubareva LV. Neuraminidase inhibitor resistance in influenza viruses and laboratory testing methods. Antiviral therapy. 2012;17(1 Pt B):159–73. 10.3851/IMP2067 22311680

[35] Lee N, Chan PK, Choi KW, Lui G, Wong B, Cockram CS, et al Factors associated with early hospital discharge of adult influenza patients. Antiviral therapy. 2007;12(4):501–8. 17668558

[36] Coffin SE, Leckerman K, Keren R, Hall M, Localio R, Zaoutis TE. Oseltamivir shortens hospital stays of critically ill children hospitalized with seasonal influenza: a retrospective cohort study. Pediatr Infect Dis J. 2011;30(11):962–6. 10.1097/INF.0b013e318232ede9 21997661 PMC3426912

[37] Heinonen S, Silvennoinen H, Lehtinen P, Vainionpaa R, Vahlberg T, Ziegler T, et al Early oseltamivir treatment of influenza in children 1–3 years of age: a randomized controlled trial. Clin Infect Dis. 2010;51(8):887–94. 10.1086/656408 20815736

[38] Ison MG, Sharma A, Shepard JA, Wain JC, Ginns LC. Outcome of influenza infection managed with oseltamivir in lung transplant recipients. J Heart Lung Transplant. 2008;27(3):282–8. 10.1016/j.healun.2007.11.575 18342750

[39] Cheung DH, Tsang TK, Fang VJ, Xu J, Chan K-H, Ip DKM, et al Association of Oseltamivir Treatment With Virus Shedding, Illness, and Household Transmission of Influenza Viruses. The Journal of infectious diseases. 2015;212(3):391–6. 10.1093/infdis/jiv058 25646354 PMC4866553

[40] Marriott AC, Dove BK, Whittaker CJ, Bruce C, Ryan KA, Bean TJ, et al Low Dose Influenza Virus Challenge in the Ferret Leads to Increased Virus Shedding and Greater Sensitivity to Oseltamivir. PLoS ONE. 2014;9(4):e94090 10.1371/journal.pone.0094090 24709834 PMC3978028

[41] Govorkova EA, Ilyushina NA, Boltz DA, Douglas A, Yilmaz N, Webster RG. Efficacy of oseltamivir therapy in ferrets inoculated with different clades of H5N1 influenza virus. Antimicrob Agents Chemother. 2007;51(4):1414–24. 10.1128/AAC.01312-06 17296744 PMC1855473

[42] Belser JA, Maines TR, Creager HM, Katz JM, Tumpey TM. Oseltamivir inhibits influenza virus replication and transmission following ocular-only aerosol inoculation of ferrets. Virology. 2015;484:305–12. 10.1016/j.virol.2015.06.020 26142497 PMC5729277

[43] Takahashi K, Furuta Y, Fukuda Y, Kuno M, Kamiyama T, Kozaki K, et al In vitro and in vivo activities of T-705 and oseltamivir against influenza virus. Antivir Chem Chemother. 2003;14(5):235–41. 14694986 10.1177/095632020301400502

[44] Kocik J, Kolodziej M, Joniec J, Kwiatek M, Bartoszcze M. Antiviral activity of novel oseltamivir derivatives against some influenza virus strains. Acta Biochim Pol. 2014;61(3):509–13. 25210935

[45] McKimm-Breschkin JL. Influenza neuraminidase inhibitors: antiviral action and mechanisms of resistance. Influenza and Other Respiratory Viruses. 2013;7(Suppl Suppl 1):25–36. 23279894 10.1111/irv.12047 PMC4942987

[46] McKimm-Breschkin J, Trivedi T, Hampson A, Hay A, Klimov A, Tashiro M, et al Neuraminidase Sequence Analysis and Susceptibilities of Influenza Virus Clinical Isolates to Zanamivir and Oseltamivir. Antimicrobial Agents and Chemotherapy. 2003;47(7):2264–72. 10.1128/AAC.47.7.2264-2272.2003 12821478 PMC161875

[47] Herlocher ML, Carr J, Ives J, Elias S, Truscon R, Roberts N, et al Influenza virus carrying an R292K mutation in the neuraminidase gene is not transmitted in ferrets. Antiviral research. 2002;54(2):99–111. 12062395 10.1016/s0166-3542(01)00214-5

[48] Bloom JD, Gong LI, Baltimore D. Permissive secondary mutations enable the evolution of influenza oseltamivir resistance. Science (New York, NY). 2010;328(5983):1272–5. 10.1126/science.1187816 PMC2913718 20522774

[49] Butler J, Hooper KA, Petrie S, Lee R, Maurer-Stroh S, Reh L, et al Estimating the fitness advantage conferred by permissive neuraminidase mutations in recent oseltamivir-resistant A(H1N1)pdm09 influenza viruses. PLoS pathogens. 2014;10(4):e1004065 10.1371/journal.ppat.1004065 24699865 PMC3974874

[50] Englund J, Feuchtinger T, Ljungman P. Viral Infections in Immunocompromised Patients. Biology of blood and marrow transplantation: journal of the American Society for Blood and Marrow Transplantation. 2011;17(1 Suppl):S2–S5. 10.1016/j.bbmt.2010.11.008 PMC3030455 21195305

