# Supplementary material for: Influenza A/H3N2 virus infection in immunocompromised ferrets and emergence of antiviral resistance
Source: PLoS One. 2018 Jul 19;13(7):e0200849. doi: 10.1371/journal.pone.0200849 (PMC6053203; doi:10.1371/journal.pone.0200849)
Supplement: S1 Fig — Four days before virus inoculation, all ferrets were given a cocktail of antibiotics (10 mg/kg amoxicillin and 2.5 mg/kg clavulanic acid) once daily till the end of the experiment. The ferrets assigned to the immunosuppressive groups (group 2 and 3) were given a mixture of immunosuppressant (20 mg/kg MMF, 0.5 mg/kg, tacrolimus and 8 mg/kg prednisolone) starting 3 days prior to virus inoculation twice daily till the end of the experiment. All ferrets were inoculated on day 0 with 104 TCID50 A/NL/16/98 (H3N2) influenza virus. One day after virus infection, OSP (10 mg/kg) was administered to ferrets from group 3 and 4. Ferrets from groups 1, 2 and 3 were euthanized 18 days post infection and the once from group 4 were euthanized 7 days post infection. (PDF) [file pone.0200849.s001.pdf]

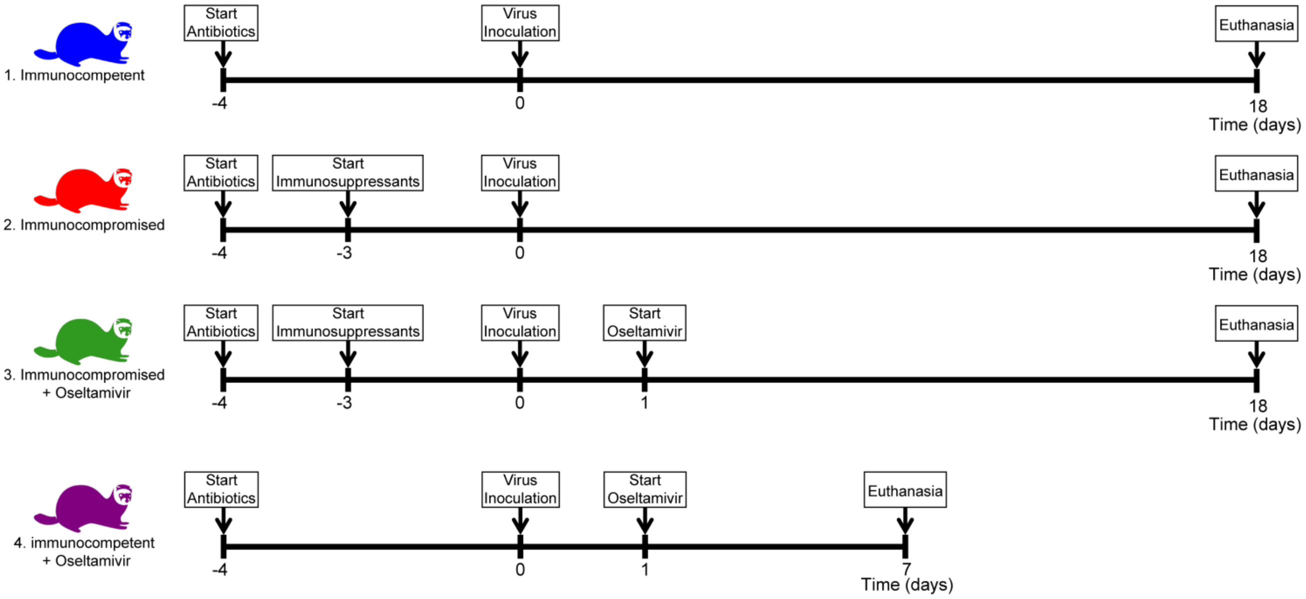

S1 Fig. Scheme of time of treatment start and virus inoculation in ferrets of the 4 experimental groups

Four days before virus inoculation, all ferrets were given a cocktail of antibiotics (10 mg/kg amoxicillin and 2.5 mg/kg clavulanic acid) once daily till the end of the experiment. The ferrets assigned to the immunosuppressive groups (group 2 and 3) were given a mixture of immunosuppressant (20 mg/kg MMF, 0.5 mg/kg, tacrolimus and 8 mg/kg prednisolone) starting 3 days prior to virus inoculation twice daily till the end of the experiment. All ferrets were inoculated on day 0 with 10^4 TCID50 A/NL/16/98 (H3N2) influenza virus. One day after virus infection, OSP (10mg/kg) was administered to ferrets from group 3 and 4. Ferrets from groups 1, 2 and 3 were euthanized 18 days post infection and the once from group 4 were euthanized 7 days post infection.
