# Supplementary material for: Influenza A/H3N2 virus infection in immunocompromised ferrets and emergence of antiviral resistance
Source: PLoS One. 2018 Jul 19;13(7):e0200849. doi: 10.1371/journal.pone.0200849 (PMC6053203; doi:10.1371/journal.pone.0200849)
Supplement: S2 Fig — Serum influenza antibody titers of the untreated immunocompetent and immunocompromised ferrets against the influenza homologous strain (A) A/NL/16/98 and (B) the heterologous strain A/NL/271/95 of immunocompetent and immunocompromised ferrets were determined by hemagglutinin inhibition (HI) assay. The antibody titer of each animal are depicted as individuals points. The horizontal bars represent the mean ± S.E.M. (PDF) [file pone.0200849.s002.pdf]

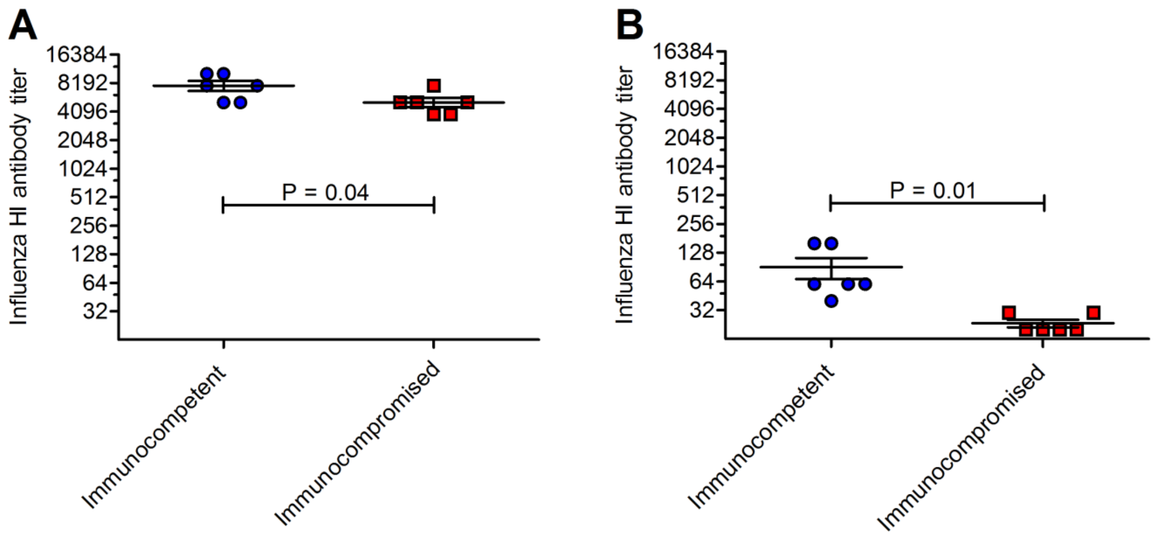

S2 Fig. Influenza antibody titers of immunocompetent and immunocompromised ferrets against influenza viruses A/NL/16/98 and A/NL/271/95.

Serum influenza antibody titers of the untreated immunocompetent and immunocompromised ferrets against the influenza homologous strain (A) A/NL/16/98 and (B) the heterologous strain A/NL/271/95 of immunocompetent and immunocompromised ferrets were determined by hemagglutinin inhibition (HI) assay. The antibody titer of each animal are depicted as individuals points. The horizontal bars represent the mean ± S.E.M.
