# Supplementary material for: Influenza A/H3N2 virus infection in immunocompromised ferrets and emergence of antiviral resistance
Source: PLoS One. 2018 Jul 19;13(7):e0200849. doi: 10.1371/journal.pone.0200849 (PMC6053203; doi:10.1371/journal.pone.0200849)
Supplement: S3 Fig — Immunocompetent (blue, purple) and immunocompromised (red, green) ferrets were inoculated with influenza virus A/NL/16/98 (H3N2) and subsequently treated with oseltamivir (green, purple) or left untreated (blue, red). Virus RNA load in samples of the throat (A) and nose (C) was determined by qRT-PCR daily for 7 days. The area under the curve of panels A and C was used to estimate the total amount of viral RNA shedding from the throat (B) and nose (D) of the inoculated ferrets. The line and bar graphs depict the mean ± S.E.M. The asterisks indicates a statistically significant P value (0.01<**P>0.001, ***P<0.001). (PDF) [file pone.0200849.s003.pdf]

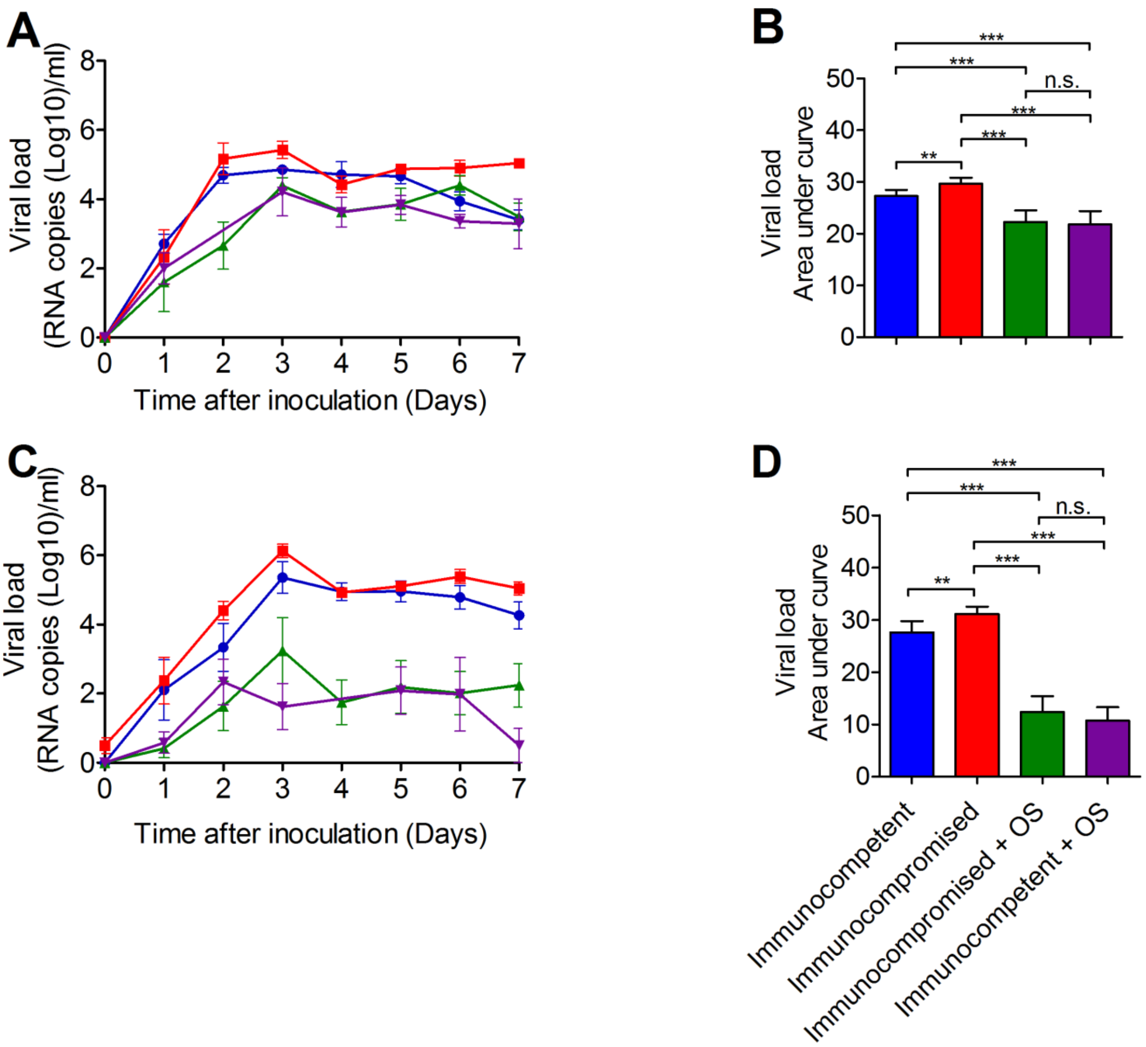

S2 Fig. Viral RNA load in ferrets inoculated with influenza virus A/NL/16/98.

Immunocompetent (blue, purple) and immunocompromised (red, green) ferrets were inoculated with influenza virus A/NL/16/98 (H3N2) and subsequently treated with oseltamivir (green, purple) or left untreated (blue, red). Virus RNA load in samples of the throat (A) and nose (C) was determined by qRT-PCR daily for 7 days. The area under the curve of panels A and C was used to estimate the total amount of viral RNA shedding from the throat (B) and nose (D) of the inoculated ferrets. The line and bar graphs depict the mean ± S.E.M. The asterisks indicates a statistically significant P value (0.01<**P>0.001, ***P<0.001).
