# Supplementary material for: Influenza A/H3N2 virus infection in immunocompromised ferrets and emergence of antiviral resistance
Source: PLoS One. 2018 Jul 19;13(7):e0200849. doi: 10.1371/journal.pone.0200849 (PMC6053203; doi:10.1371/journal.pone.0200849)
Supplement: S4 Fig — The emergence of oseltamivir resistant viruses was monitored in throat and nose samples from untreated immunocompetent ferrets (A), untreated immunocompromised ferrets (B), oseltamivir treated immunocompromised ferrets (C) and treated immunocompetent ferrets (D) by mutation-specific RT-PCR for the oseltamivir-resistance mutation R292K. The green bars depict the presence of the wildtype genotype (292R) and the magenta the presence of the resistant genotype (292K). If both genotypes were present in one sample, the proportions of the genotypes are stacked. (PDF) [file pone.0200849.s004.pdf]

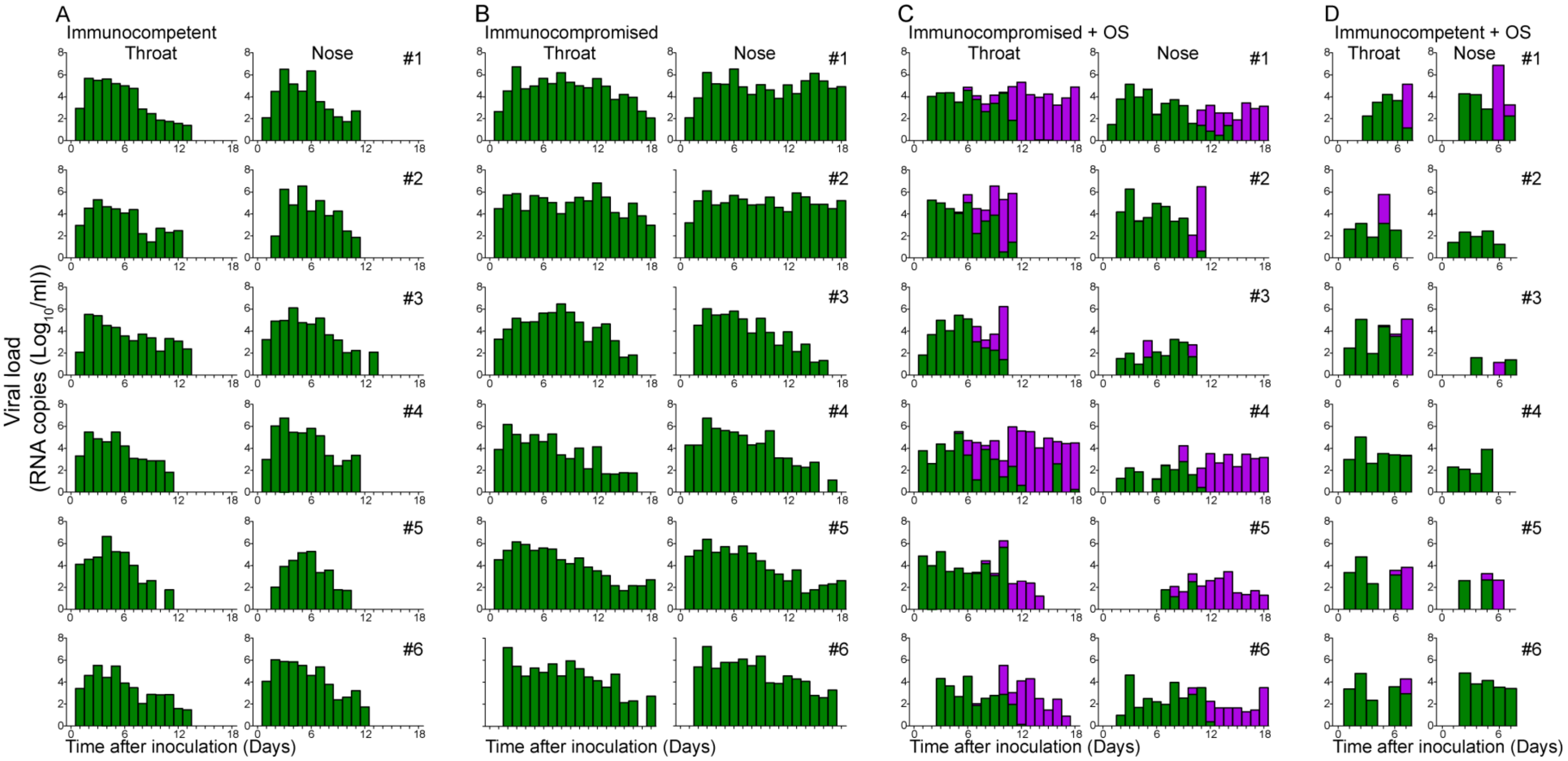

S3 Fig. Emergence of oseltamivir resistant viruses in ferrets.

The emergence of oseltamivir resistant viruses was monitored in throat and nose samples from untreated immunocompetent ferrets (A), untreated immunocompromised ferrets (B), oseltamivir treated immunocompromised ferrets (C) and treated immunocompetent ferrets (D) by mutation-specific RT-PCR for the oseltamivir-resistance mutation R292K. The green bars depict the presence of the wildtype genotype (292R) and the magenta the presence of the resistant genotype (292K). If both genotypes were present in one sample, the proportions of the genotypes are stacked.
