# Supplementary material for: Influenza A/H3N2 virus infection in immunocompromised ferrets and emergence of antiviral resistance
Source: PLoS One. 2018 Jul 19;13(7):e0200849. doi: 10.1371/journal.pone.0200849 (PMC6053203; doi:10.1371/journal.pone.0200849)
Supplement: S5 Fig — The relative proportion of the R292K resistance mutation detected in samples of all oseltamivir treated immunocompromised (grey circles) and immunocompetent (open squares) ferrets inoculated with A/NL/16/98 (H3N2) as determined by mutation specific RT-PCR was compared to the relative proportion determined by Illumina NGS. (PDF) [file pone.0200849.s005.pdf]

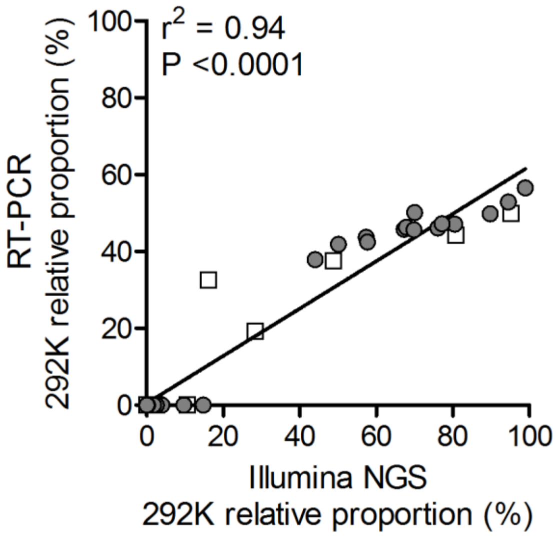

S4 Fig. Correspondence between the R292K resistance mutation as measured by mutation specific RT-PCR and Illumina next generation sequencing.

The relative proportion of the R292K resistance mutation detected in samples of all oseltamivir treated immunocompromised (grey circles) and immunocompetent (open squares) ferrets inoculated with A/NL/16/98 (H3N2) as determined by mutation specific RT-PCR was compared to the relative proportion determined by Illumina NGS**.**
