# Supplementary material for: Influenza A/H3N2 virus infection in immunocompromised ferrets and emergence of antiviral resistance
Source: PLoS One. 2018 Jul 19;13(7):e0200849. doi: 10.1371/journal.pone.0200849 (PMC6053203; doi:10.1371/journal.pone.0200849)
Supplement: S6 Fig — The percentage of the R292K variant was determined in pooled throat/nose samples collected at 2, 4, 6, 8 and 10 dpi from immunocompetent ferrets that received oseltamivir treatment. The graph depicts the frequency of the R292K mutation over time for each ferret separately, with circles, squares, triangles, down triangles, diamonds and open circles representing ferrets 1–6 respectively. (PDF) [file pone.0200849.s006.pdf]

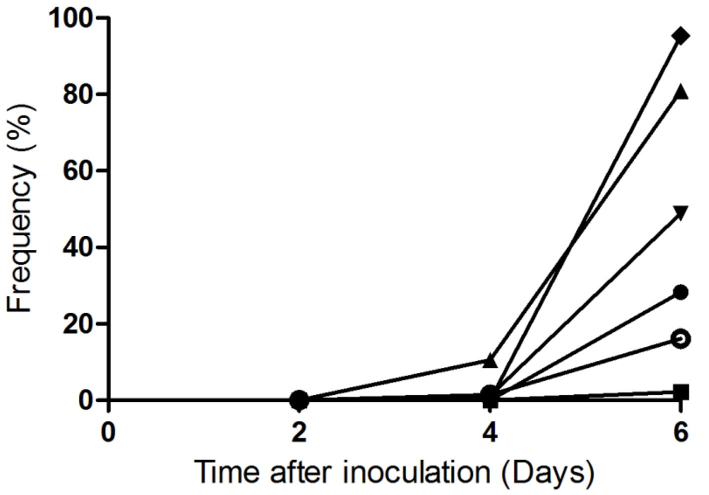

S5 Fig. The frequency of the R292K mutation as determine by Illumina next generation sequencing in oseltamivir treated immunocompetent ferrets.

The percentage of the R292K variant was determined in pooled throat/nose samples collected at 2, 4, 6, 8 and 10 dpi from immunocompetent ferrets that received oseltamivir treatment. The graph depicts the frequency of the R292K mutation over time for each ferret separately, with circles, squares, triangles, down triangles, diamonds and open circles representing ferrets 1-6 respectively.

Tables

S1 Table: Amino acid substitution in hemagglutinin and neuraminidase sequences of OS treated immunocompetent ferrets by Illumina next-generation sequencing (NGS).

Ferret	Day[a]	R292K (%)	AA[b] change in HA[b] (%)	AA change in NA[b] (%)
**1**	2	0	D204G(5.0), E442G(1.4), G442D(1.1)	
	4	0		
	6	27.1		
**2**	2	0	K18R(5.0), Q49R(1.3), S61N(1.3), T64I(1.3), G65D(1.3), C493TY(2.6)	I464L(1.9)
	4	0		
	6	2.2		L81P(1.5), T325I(2.2)
**3**	2	0	R2470W(1.3), E473G(1.2), F483S(1.0), D503G(1.4)	S367G(1.0)
	4	15.9	E442G(5.3), A475T(2.7), E476Q(2.7), L543F(7.7)	F22S(21.4), V360M(5.2)
	6	78.5		V360M(0.8)
**4**	2	0		
	4	0	N97D(8.8), N338I(1.0), G358S(1.6), Q372L(1.4), E522G(2.0)	K75N(2.6), D113E(2.2)
	6	48	Y318F(1.1)	
**5**	2	0		
	4	0	I377R(1.3)	S245G(4.8), I464L(1.8)
	6	92.0		

**6**	2	0		
	4	0	A122V(1.0), E442G(2.8), Y502H(1.3), D503Y(1.3)	
	6	17.5	A269T(3.7), I355R(4.4), E510G(2.0)	G137E(3.3), R210G(2.8), Y453C(1.2)

[a] Time post infection when resistance mutations is detected.

[b] Abbreviation: AA, amino acid; HA, hemagglutinin; NA, neuraminidase.
