# Supplementary material for: Influenza A/H3N2 virus infection in immunocompromised ferrets and emergence of antiviral resistance
Source: PLoS One. 2018 Jul 19;13(7):e0200849. doi: 10.1371/journal.pone.0200849 (PMC6053203; doi:10.1371/journal.pone.0200849)
Supplement: S1 Table — (PDF) [file pone.0200849.s007.pdf]

S1 Table: Amino acid substitution in hemagglutinin and neuraminidase sequences of OS treated immunocompetent ferrets by Illumina next generation sequencing.

Ferret	Day[a]	R292K (%)	AA[b] change in HA[b] (%)	AA change in NA[b] (%)
**1**	2	0	D204G(5.0), E442G(1.4), G442D(1.1)	
	4	0		
	6	27.1		
**2**	2	0	K18R(5.0), Q49R(1.3), S61N(1.3), T64I(1.3), G65D(1.3), C493TY(2.6)	I464L(1.9)
	4	0		
	6	2.2		L81P(1.5), T325I(2.2)
**3**	2	0	R2470W(1.3), E473G(1.2), F483S(1.0), D503G(1.4)	S367G(1.0)
	4	15.9	E442G(5.3), A475T(2.7), E476Q(2.7), L543F(7.7)	F22S(21.4), V360M(5.2)
	6	78.5		V360M(0.8)
**4**	2	0		
	4	0	N97D(8.8), N338I(1.0), G358S(1.6), Q372L(1.4), E522G(2.0)	K75N(2.6), D113E(2.2)
	6	48	Y318F(1.1)	
**5**	2	0		
	4	0	I377R(1.3)	S245G(4.8), I464L(1.8)
	6	92.0		
**6**	2	0		
	4	0	A122V(1.0), E442G(2.8), Y502H(1.3), D503Y(1.3)	
	6	17.5	A269T(3.7), I355R(4.4), E510G(2.0)	G137E(3.3), R210G(2.8), Y453C(1.2)

[a] Time post infection when resistance mutations is detected.

[b] Abbreviation: AA, amino acid; HA, hemagglutinin; NA, neuraminidase.
